# Selenium nanoparticle-enriched *Lactobacillus brevis* causes more efficient immune responses in vivo and reduces the liver metastasis in metastatic form of mouse breast cancer

**DOI:** 10.1186/2008-2231-21-33

**Published:** 2013-04-30

**Authors:** Mohammad Hossein Yazdi, Mehdi Mahdavi, Neda Setayesh, Mohammad Esfandyar, Ahmad Reza Shahverdi

**Affiliations:** 1 Department of Pharmaceutical Biotechnology, Faculty of Pharmacy and Biotechnology Research Center, Tehran University of Medical Sciences, Tehran, Iran; 2 Department of virology, Pasteur Institute of Iran, Tehran, Iran

**Keywords:** Selenium enriched *L. brevis*, Immune responses, 4T1 breast cancer, Liver metastasis

## Abstract

**Background and the purpose of the study:**

Selenium enriched *Lactobacillus* has been reported as an immunostimulatory agent which can be used to increase the life span of cancer bearing animals. Lactic acid bacteria can reduce selenium ions to elemental selenium nanoparticles (SeNPs) and deposit them in intracellular spaces. In this strategy two known immunostimulators, lactic acid bacteria (LAB) and SeNPs, are concomitantly administered for enhancing of immune responses in cancer bearing mice.

**Methods:**

Forty five female inbred BALB/c mice were divided into three groups of tests and control, each containing 15 mice. Test mice were orally administered with SeNP-enriched *Lactobacillus brevis* or *Lactobacillus brevis* alone for 3 weeks before tumor induction. After that the administration was followed in three cycles of seven days on/three days off. Control group received phosphate buffer saline (PBS) at same condition. During the study the tumor growth was monitored using caliper method. At the end of study the spleen cell culture was carried out for both NK cytotoxicity assay and cytokines measurement. Delayed type hypersensitivity (DTH) responses were also assayed after 72h of tumor antigen recall. Serum lactate dehydrogenase (LDH) and alkaline phosphatase (ALP) levels were measured, the livers of mice were removed and prepared for histopathological analysis.

**Results:**

High level of IFN-γ and IL-17 besides the significant raised in NK cytotoxicity and DTH responses were observed in SeNP-enriched *L. brevis* administered mice and the extended life span and decrease in the tumor metastasis to liver were also recorded in this group compared to the control mice or *L.brevis* alone administered mice.

**Conclusion:**

Our results suggested that the better prognosis could be achieved by oral administration of SeNP-enriched *L. brevis* in highly metastatic breast cancer mice model.

## Introduction

Using the immunomodulatory agents in the field of cancer treatment has a growing trend during last decades [1,2]. This strategy is also known as adjuvant therapy and some studies have demonstrated the advantages of this strategy for cancer treatment [3]. Immunomodulatory agents are divided in two groups of immunosupressors and immunostimulators. Chemical immunomodulators like levamizole have been used in order to stimulate immune response against cancer and AIDS [4]. Natural immunomodulators like glucan or other oligosaccharide components which are mostly derived from microbial source are being applied to stimulate the immune response against cancers [5].

Lactic acid bacteria (LAB) are Gram positive normal flora which shows variety of beneficial effects on their host’s health including the prevention of tumor growth [6]. Many studies which include the effects of orally administered lactobacilli and bifidobacteria on the immune system have been performed in animal models (e.g., tumor, infection, and allergy models) [7,8]. An increase in natural killer (NK) cell activity was observed in mice which were administered orally with some strains of LAB [9]. The activation of the systemic and secretary immune response by LAB requires many complex interactions among the different constituents of the intestinal ecosystem (microflora, epithelial cells and immune cells). It also seems that some of these immunological effects may be related to the cell components of LAB bacteria [10]. As many studies have demonstrated it is notably interesting that immune system can be optimized through oral supplementation of specific *Lactobacillus* strains [11,12]. Depending on their intuitive properties, orally applied lactobacilli have been reported to affect T-helper 1 and T-helper 2 pathways by local cytokine production in the gut and systemic specific antibody formation [13,14].

On the other hand selenium (Se), as an essential micronutrient element which exhibit anticarcinogenic effects, can prevent the transformation of normal cells to malignant cells and the activation of oncogenes in transformed cells [15,16]. Consumption of Se affects the development and expression of nonspecific, humoral, and cell-mediated immune responses and deficiency in Se appears to result in immunosuppression, whereas supplementation with low doses of Se appears to result in augmentation or restoration of immunologic functions [17]. SeNP-enriched *Lactobacillus* can be considered as a new form of Se organic products. Recently we reported the effect of SeNP-enriched *Lactobacillus plantarum* on the immune response and lifespan of 4T1 breast cancer bearing mice and show this can increase host immune response (i.e. NK cell cytotoxicity) and enhanced the survival rate of animals for 130 days [18]. To obtain a better survival rate, in the current study we isolated and characterized another immunostimulant lactic acid bacterium (*Lactobacillus brevis*) and used for intracellular reduction of Se ions. These SeNP-enriched probiotic has been administered in mice bearing 4T1 breast cancer tumor and the effect of this administration on the survival of cancerous animals, immune responses and liver metastasis was compared to *L. brevis* alone.

## Materials and methods

### Animals

Forty five female inbred BALB/c mice with six to eight weeks old and each weighing from 25 to 30 g, were purchased from the Pasture Institute of Iran (Tehran, Iran). They were divided into three groups of test and control, each containing 15 mice. The mice were kept in plastic cages, allowed free access to water, and maintained on a 12:12 h light and dark cycle during the study period. The temperature and humidity were controlled at 23 ± 1°C and 55 ± 10%, respectively, and all mice were fed via a standard mouse pellet diet. The control mice in this study were kept separated from the test group, but at the same temperature and humidity, and they were fed the same food.

### Lactobacillus isolation and characterization

The Lactobacillus bacterium was isolated from human feces and characterized by 16s rDNA sequence analysis [19]. Genomic DNA was extracted from bacterial cells using PrimePrep Genomic DNA isolation kit according to the manufacturer’s instructions. It was then subjected to PCR amplification using universal primers 27F (5′ GAGTTTGATCCTGGCTCAG-3′) and 1492R (5′-GGTTACCTTGTTACGACTT-3′) targeting the conserved regions of bacterial 16S ribosomal RNA gene. The amplification program consisted of one cycle of 94°C for 3 min; 30 cycles of 94°C for 20 s, 55 for 30 s, and 72°C for 2 min; and finally one cycle of 72°C for 5 min. The amplified DNA fragment was purified from 1% agarose gel using the QIAquick Gel Extraction Kit (Qiagen, USA) according to the supplier's instructions and was sent for automated sequencing using the above primers (GenFanAvaran Co., Iran). Sequence similarity searches were done with the BLAST database (National Center for Biotechnology Information), and the sequence was submitted to GenBank.

### Preparation of SeNP-enriched and non-enriched probiotics

The isolated LAB bacterium which was identified as *Lactobacillus brevis* was inoculated into 10 ml of DeMan–Rogosa–Sharpe (MRS) broth (Merck, Germany) and cultivated overnight at 37°C under anaerobic conditions. One mL of a stock solution of SeO_2_ (254 mM) was then added to 100 ml of *Lactobacillus* broth culture to reach a final concentration of 200 mg/L Se ions (corresponding to a 2.54 mM solution of SeO_2_), and incubation at 37°C was continued for 72h. During this time, the Se ions were reduced to form intracellular red elemental selenium. The bacteria were then collected by centrifugation at 4000 × g for 30 min at 4°C, washed three times with sterile phosphate buffer saline (PBS), and used for the animal study.

### Animal study

The SeNP-enriched *L. brevis* alone was separately re-suspended in PBS solutions to obtain the desired bacterial cell concentration of 2.7 × 10^8^ CFU/ml. 0.5 ml of this suspension (containing about 100 μg/ml of Se ions) was orally administered to the mice by using a standard gastric feeding gavage as follows:

The test group of mice was daily administered with 0.5 ml of SeNP-enriched *L. brevis* suspension for three consecutive weeks prior to tumor challenge. This daily feeding was continued for three repeated cycles of seven days on/three days off. Same above protocol was also concomitantly repeated for *L .brevis* alone. Also the control mice (PBS-received mice) were given an equal volume of PBS solution in a similar procedure.

### Tumor challenge

The 4T_1_ cell line ATCC CRL-2539 (originated from mice breast tumor and is routinely applied to simulate the end stage of human breast cancer in animal model) was used for the induction of tumors in inbred Balb/c mice. For this purpose, 200 μl of RPMI, containing 4T_1_ cells at a concentration of 1 × 10^6^ cell/ml, was injected subcutaneously near the mammary glands of female mice. All mice were followed until a tumor nodule was observed (10 days after injection).

### The tumor growth measurement

Tumor growth was measured twice during this study firstly when tumors became palpable and at last before mice scarification by caliper measurement of the tumor length in two different dimensions. The tumor volumes of 15 mice in each test and control groups were determined using the formula: V = 0.5×d^2^×D [20] where V is the tumor volume (mm^3^), d is the shorter diameter, and D is the longer diameter. Finally average tumor volumes of last time measurement were subtracted from the first time and reported as related tumor volume.

### Providing the tumor antigen

The tumor antigen was prepared from the tumor of one of the mice. The tumor was removed from the body, dissected into small sections (1 mm^3^), and homogenized in sterile PBS. This homogenized sample was washed with sterile PBS, 100 μl/ml of PMSF was added to inhibit the endogenous proteases, and the sample was sonicated (Hielscher Ultrasonics GmbH, Germany) for 10 minute. The sample was then dialyzed against 1000 ml of PBS buffer for 24 h at 4°C using a cellulose membrane with a 14 kD cutoff (Sigma, Germany) and the PBS was changed every eight hours. After dialysis, the concentrated sample was collected and the protein concentration was determined using the conventional Bradford method. The sample was used to stimulate splenocytes in the cytokine production tests and delayed type hypersensitivity response (DTH) assay.

### Evaluation of DTH response

The DTH response was measured according to a method described by Jin et al. [21]. Briefly, 30 days after tumor challenge, 7 mice from each group were challenged with the tumor antigen (20 μg) in the left footpad and with the PBS in the right footpad. Footpad induration was measured at 72 h later using caliper measurements.

### Cytokine determination in spleen cell culture

One month after tumor challenge, spleens of eight mice from each group were removed aseptically and dissected. Spleen cells were then prepared using an appropriate nylon mesh screen and the remaining RBCs in the spleen cell collection were disrupted with conventional RBC lysis buffer. The spleen cell counts were adjusted to 2.5 × 10^6^ cell/ml in RPMI1640 (Gibco Life Technologies, Germany) supplemented with 10% fetal bovine serum (Invitrogen, Paisley Germany), 100 μg/ml streptomycin, and 100 IU/ml penicillin (Sigma, Germany). These cells were then stimulated with 20 μg/ml of the tumor antigen (the amount was determined in a previous study) for 72 h [4] at 37°C in a humidified atmosphere of 5% CO_2_. The levels of IL-17 and IFN-γ in the spleen cell culture supernatants were determined using ELISA kits (R&D Systems, Minneapolis, MN), according to the manufacturer’s instructions. The limit of detection for IFN-γ was 2 pg and for IL-17 was 5 pg.

### Natural killer cell activity by LDH assay

The NK cell activity was evaluated in eight mice from each group (these mice were also used for the cytokine determination test). An aliquot containing 2.5 × 10^6^ splenocytes was removed and mixed with target K562 cells at a ratio of 1:100 (Target:Effector) in 96-well culture plates with U-shaped bottoms (Corning, Corning, NY) in 0.2 ml of RPMI1640 containing 2% BSA. The release of LDH was measured using an LDH assay kit (Takara, Japan) by first gently centrifuging the plates for 5 min at 250 × g and then incubating them for four h at 37°C in 5% CO_2_. After incubation, the plates were centrifuged for 10 min at 250 × g, and the supernatant was transferred from each well of the culture plate to the corresponding well of a new 96 well plate. One hundred microliters of reaction solution (prepared according to the kit instructions) was then added to each well, and the plate was incubated with gentle shaking on an orbital shaker for 30 min. The absorbance of each well was then read at 490 nm. The specific release of LDH was calculated as a percentage using the following formula [22]:

Experimentalvalue−lowcontrol/highcontrol−lowcontrol×100

### LDH and ALP determination in blood circulation

Blood was collected from pre-orbital cavity of eight mice from each group (these mice were also used for spleen cell culture). Each mouse was placed under the anesthesia induced by ether and the blood samples (one ml) were collected from the pre-orbital cavity into appropriate microtubes. Serum samples were prepared by storing the blood at 4°C for one hour, and the resulting coagulated blood cells were then separated from the serum by centrifugation for 20 min at 4000 × g. The serum was transferred to a new microtube, and the lactate dehydrogenase (LDH) and alkaline phosphatase (ALP) levels in each sample was determined using the IFCC method [23,24].

### Histopathological studies

After cervical dislocation of experimental mice (these mice were also used for spleen cell culture) the liver was taken for histopathological analysis. Tissues were fixed in 10% formalin in PBS solution, in embedded paraffin, and cut into 3-5-μm thick sections. The sections were stained with hematoxylin eosin for general analysis using light microscope with 400× magnification. Pathological assay was done after preparation of H&E stained slides from tumor and liver tissues. In next step, the 10 microscopic fields in each slide were carefully observed and proportions (%) of metastasis and necrosis cells were estimated from microscopic evaluation of the mentioned selected fields.

### Survival rate

At the end of the study period (one month after tumor challenge), seven mice from each group (these mice were also used for the DTH assay) were kept in standard conditions and fed with standard diet, with free access to water, and maintained under a 12:12 h light dark cycle until they died. Daily deaths were recorded; after the last death in both groups, the data were analyzed with a Kaplan-Meier test.

### Statistical analysis

All of the statistical analyses, except for survival rate, were conducted using SPSS software (Version 15.0) and ANOVA tests. The survival rate data were analyzed by the Kaplan-Meier test using also SPSS software (Version 15.0). The values are presented as mean ± SD.

### Ethical approval

The experimental procedures carried out in this study were in compliance with the guidelines of the Tehran University of Medical Science (Tehran, Iran) for the care and use of laboratory animals.

## Results

### Identification of the microorganism

BLAST search of the 16S rDNA sequence against the NCBI Nucleotide database was conducted for identification of isolate. Alignment results containing 180 characters revealed 100% identity with *L. brevis*. The 16S rRNA gene sequence of the *L. brevis* isolate has been submitted to GenBank under accession number JX966418.

### The tumor volumes growth measurement and DTH response

**Figure 1:**
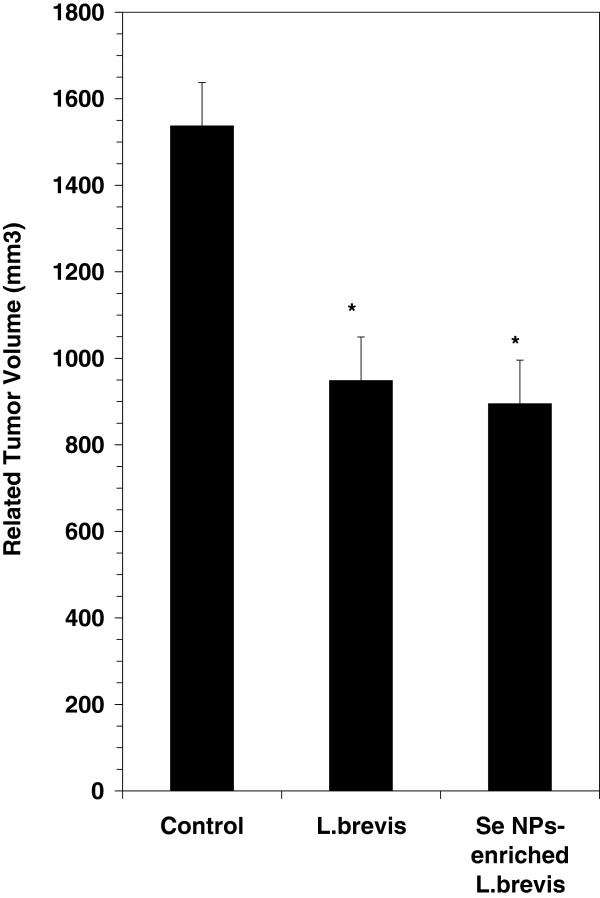
**Related tumor volume in tumor-bearing mice which received SeNP-enriched *L. brevis*, *L. brevis *alone and PBS buffer (control group).** Tumor growth was measured twice a week and evaluated by caliper measurement of the tumor length. The tumor related volume which refers to the measurement of 15 tumors in each group was achieved by subtraction of first measurement from the latest. Asterisks indicate statistical significance (*P* ≤ 0.05 significance).

**Figure 2:**
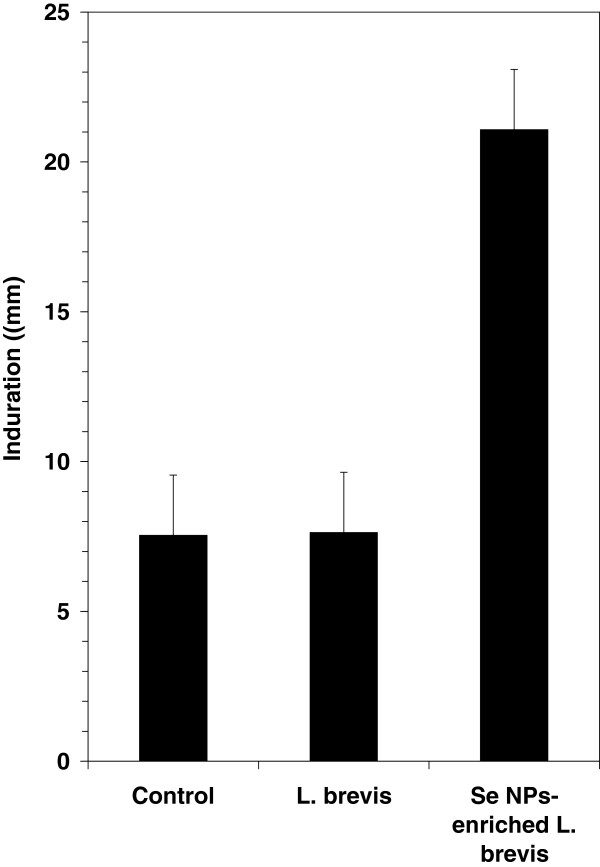
**Foot pad induration in tumor-bearing mice which received SeNP-enriched *L. brevis*, *L. brevis *alone and PBS buffer (control group).** Footpad induration was measured at 72 h after tumor antigen re-challenge using caliper measurements.

Tumor growth was evaluated by twice-weekly caliper measurements of the tumors from 15 mice from each group. The tumor volume was calculated as described in the Materials and Methods section. Data analysis showed a significant (*P* ≤ 0.05) decrease in the growth rate of tumors in the test mice when compared to the control mice (Figure 1). The tumor mass has been enlarged to 1550 mm^3^ in control mice while in test groups, which received SeNP-enriched *L. brevis* or *L. brevis* alone, the tumor volumes were decreased to 890 and 950 mm^3^, respectively. Also the antigen-specific Th1 recalling response was assessed by evaluating the DTH reaction in the tumor antigen rechallenged mice. Also as mentioned before, these mice were challenged with tumor antigen in the left footpad and with PBS solution in the right footpad 30 days after tumor injection. The results showed a greater swelling and thickness in the left footpad 72h after the tumor antigen challenge in the test group which received SeNP-enriched *L. brevis* compared with the other groups (Figure 2). In contrast, no significant increase in DTH response was observed in those test mice which received *L. brevis* alone compared to control mice. It may show that the prescription of *L. brevis* alone could not significantly enhance the Th1 recalling response.

### Cytokine determination in spleen cell culture

**Figure 3:**
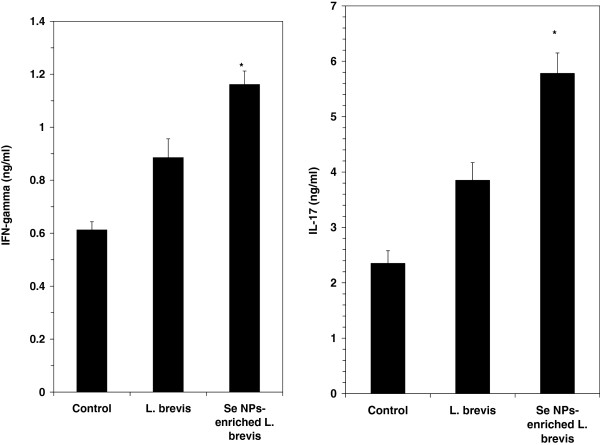
**Inductions of IFN-γ (left illustration) and IL17 (right illustration) by administration of SeNP-enriched *L. brevis *in a spleen cell culture.** The spleen cells were stimulated with tumor antigen for 72 h and then the levels of IFN-γ and IL-17 in the culture supernatants were determined by ELISA. Data represent the means ± standard deviations for triplicate cultures of eight animals per group * (*P* ≤ 0.05).

The levels of IFN-γ and IL-17 in the spleen cell culture supernatants were measured using a sandwich ELISA assay (R&D Systems, Minneapolis, MN). As shown in right illustration of Figure 3, the level of IFN-γ was significantly higher in the test groups than in the control group (*P* ≤ 0.05). The IL-17 level in the test groups was also significantly elevated when compared to the control level (Left illustration of Figure 3).

### Natural killer cell activity by LDH assay

**Figure 4:**
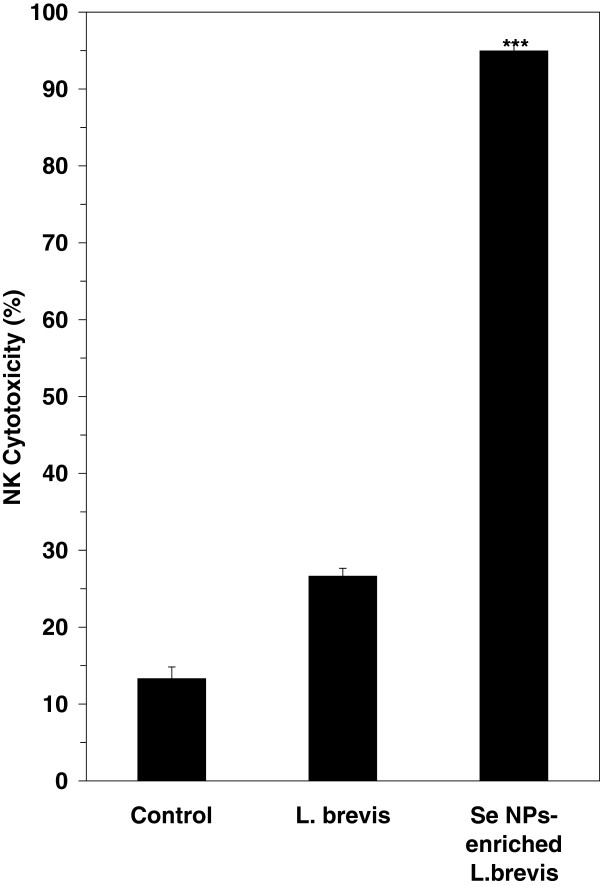
**NK cell activity in spleen cell cultures measured by the LDH assay.** A culture of 2.5 × 10^6^ cells/well from the splenocytes was added to K562 cells at a 1:100 ratio (Effector: Target ratio) and plates were gently centrifuged for five min at 50 × g, then cultured in RPMI1640 containing 10% FBS. The plates were incubated for seven h at 37°C in 5% CO_2_ and the specific release of LDH was calculated as a percentage. A significant increase * * * (*P* ≤ 0.001) in the NK cytotoxicity was observed for the SeNP-enriched *L. brevis* group compared to the control group. The data are the means ± SD of triplicate cultures. SeNP-enriched *L. brevis* = selenium nanoparticle enriched *Lactobacillus brevis.*

We investigated and compared the effects of administration of SeNP-enriched *L. brevis* and *L. brevis* alone on NK cells by using K562 cells as target cells and evaluating the release of LDH from these cells after 4 h of exposure to the NK cells that were present among the harvested splenocytes. The mice treated with SeNP-enriched *L. brevis* showed a significantly increased level of NK cell activity (*P* ≤ 0.001) compared to other groups (Figure 4) which received probiotic alone or PBS solution (control mice).

### Serum levels of LDH and ALP

**Figure 5:**
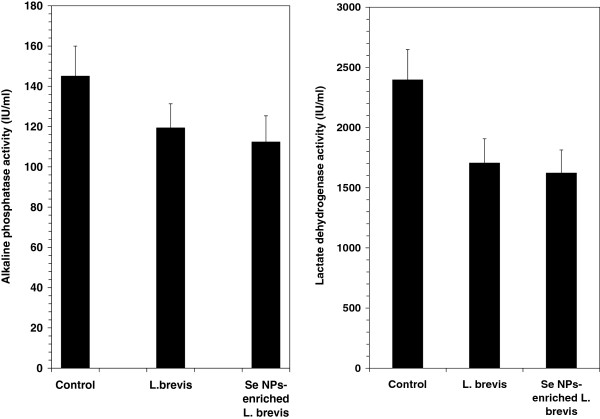
**Differences between the levels of ALP and LDH in the serum of mice, measured using the conventional IFCC method.** Data are shown in the range of U/L.

Analysis of serum showed a decrease in the levels of LDH and ALP in the mice which received SeNP-enriched *L. brevis* when compared to the other groups received *L. brevis* alone or PBS solution (control mice). The decrease in serum LDH may have directly contributed to the decrease in the rate of tumor development and tumor cell division in the groups treated by SeNP-enriched *L. brevis* or *L. brevis* alone (left illustration of Figure 5). Also the decrease in the ALP level in test mice shows better liver prognosis in both treated groups compared to control group (right illustration of Figure 5).

### Histopathological studies

**Figure 6:**
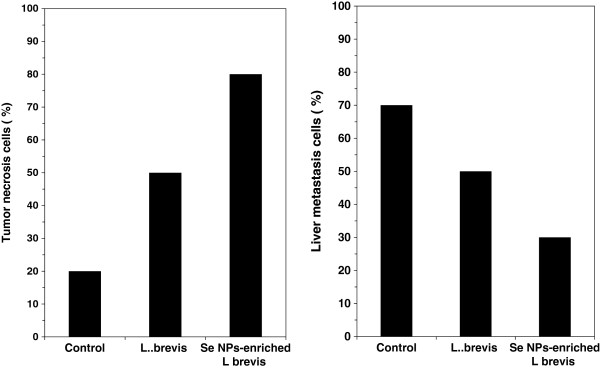
**The levels of tumor necrosis (left illustration) and liver metastasis (right illustration) cells were diagnosed in histopathological slides prepared from tissue samples removed mice which received SeNP-enriched *L. brevis*.**

As it observes in left illustration of Figure 6 the rate of tumor necrosis in test mice which received SeNP-enriched *L. brevis* was considerably increased in comparison to other test or control groups. On the other hand the highest decreasing rate in the level of liver metastasis was diagnosed in histopathological slides (Figures not shown) prepared from liver tissue of mice which received SeNP-enriched *L. brevis* (right illustration of Figure 6).

### Survival rate

**Figure 7:**
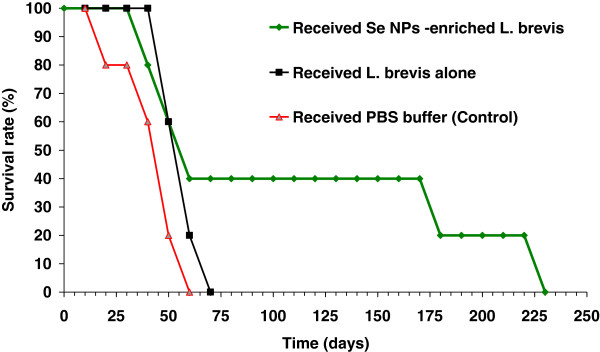
**Survival rates of mice administered SeNP-enriched *L. brevis *and *L. brevis *alone when compared with control mice at the end of the study.** A total of seven mice from each experimental group were kept under standard conditions until they died. The rate of death was registered every day and the obtained data were analyzed with a Kaplan-Meier test after the last death in both test and control groups. The lifespans of animals that received SeNP-enriched *L. brevis* or *L. brevis* alone were considerably increased compared to the lifespans of control mice.

The results of survival analysis in Figure 7 show the decrease in the rate of mortality among the SeNP-enriched *L. brevis* administered mice group in comparison to other groups. In the test group which received SeNP-enriched *L. brevis*, it was observed a remarkable decrease in mortality rate and indicated that administration of this formulation could enhance the survival rate of tumor bearing mice over 230 days period. The effect of *L. brevis* without SeNPs was also investigated on the survival rates of tumor bearing mice. Oral administration of *L. brevis* alone also enhanced the survival rate of 4T1 tumor bearing mice over 75 days period, but this enhancement was not as same as the survival rate observed in the mice treated with SeNP-enriched *L. brevis* (230 days). This result even is more considerable than the survival time of cancerous mice which received purified biogenic SeNPs (90 days) [25] or SeNP-enriched *L. plantarum* (130 days) [18].

## Discussion

Tumor development in cancer patients has been reported to attenuate immunological response in cancer bearing patients [26]. Also routine chemotherapy and irradiation treatment can considerably decrease the host immunity and are main reasons to weak immune system in cancer bearing patients [27] attenuate immunological response in cancer bearing patients. It also weakens the natural defense against foreign threats such as pathogenic or opportunistic microorganisms. Therefore using some immunomodulatory agents in immunosuppressed patients seems to be helpful to decrease the cancer development and prevent additional complications such as infectious diseases. On the other hand, metastasis is major cause of death among cancer bearing patient and can worsen the prognosis of cancer especially for those who are suffering from advanced level of cancer.

Recently we reported the immunostimulatory effect of purified biogenic SeNPs [25] and SeNP-enriched *L. plantarum* on the life span of 4T1 breast cancer animal model [18]. Both formulations have increased the survival rate of cancerous animals but the effect of SeNP-enriched *L. plantarum* on the survival rate of 4T1 cancer bearing mice was more remarkable than purified biogenic SeNPs or *L. plantarum* alone [18]. Oral administration of SeNP-enriched *L. plantarum* delayed the mortality of some cancerous animals for 130 days which was 40 days longer than the survival time of animals received purified biogenic SeNPs. Although the immunomodulatory effect of LAB is currently established [28,29] but still it should not be ignored that this effect is variable in different species of these bacteria [30]. So the oral supplementation with other SeNPs LAB strain may lead to obtain a better survival rate in cancerous animals. Regarding to above fact, in this study another strain of LAB was isolated from human feces and identified as *L. brevis*. In next step this bacterium was enriched by elemental Se and used for oral supplementation of breast cancer bearing animals. Then the effect of this type of supplementation and supplementation of *L. brevis* alone on the immune responses and the liver metastasis have been investigated and compared in breast cancer bearing animals.

SeNP-enriched *Lactobacillus* is an organoselenium agent and a new form of Se which is synthesis and accumulated in intracellular space of bacteria, this form of Se also considered as biogenic SeNPs [31]. Although results of some studies implied to the protective effect of Se enriched *lactobacillus* on the CCL_4_ liver injury but as a best of the authors’ knowledge it still no more understood about immunostimulatory effect of this new form of LAB [32]. Cytokine assay in the current study showed an increase in the level of IFN-γ through the oral administration of SeNP- enriched *L. brevis*. On the other hand IL-17 as another Th1 known cytokine also raised due to this administration. Moreover, NK cell activity was enhanced in the test mice which received SeNP-enriched *L. brevis* and could be related to the increase in the level of IFN-γ in some way.

NK cells play a major role in the rejection of tumors and cells infected by viruses [32,33]. Also any decrease in tumor volume occurred in SeNP-enriched *L. brevis* administered mice can address to engaging of antitumor immune responses such as NK cytotoxicity (Figure 4). Enhancement of tumor necrosis as well as higher DTH response in test mice which received SeNP- enriched *L. brevis* can further confirm this hypothesis (Figure 2).

In the other hand, although the serum levels of aspartate aminotransferase (AST) and alanine aminotransferase (ALT) as two types of liver function enzymes were similar in test and control groups (data are not shown) but regarding to the result of histopathological study the tumor metastasis into liver of SeNP-enriched *L. brevis* treated mice was considerably lower than control group which received PBS buffer. Moreover, it observed that by administration of SeNP- enriched *L. brevis* the serum level of ALP and LDH, which are considered for cancer monitoring, have been reduced. Conclusively, the enhancement of life span in SeNP- enriched *L. brevis* administered mice which observed during this investigation as a promising result can introduce this oral formulation as a good candidate for future prevention and immunotherapy of cancer. But still more studies are needed to develop this formula.

## Competing interests

The authors declare that they have no competing interests.

## Authors’ contribution

YMH: Conducting experiments and manuscript preparation, MM: Supervising Immunoassay experiments, NS: Conducting molecular experiment, ME: Participating in animal experiments, ShAR: Project design, supervising experiments and manuscript preparation. All authors read and approve the final manuscript.
